# Problematic Smartphone Use in Adolescents with Attention-Deficit/Hyperactivity Disorder: The Roles of Domestic Violence, Parenting Styles, and Peer Bullying Victimization

**DOI:** 10.7150/ijms.112302

**Published:** 2025-06-23

**Authors:** Po-Chun Lin, Cheng-Fang Yen, Ray C. Hsiao, Peng-Wei Wang

**Affiliations:** 1 Department of Psychiatry, E-Da Hospital, Kaohsiung, Taiwan.; 2 School of Medicine, College of Medicine, I-Shou University, Kaohsiung 82445, Taiwan.; 3 Department of Psychiatry, School of Medicine, College of Medicine, Kaohsiung Medical University, Kaohsiung, Taiwan.; 4 Department of Psychiatry, Kaohsiung Medical University Hospital, Kaohsiung, Taiwan.; 5 College of Professional Studies, National Pingtung University of Science and Technology, Pingtung, Taiwan.; 6 Department of Psychiatry, Seattle Children's, Seattle, WA, USA.; 7 Department of Psychiatry and Behavioral Sciences, School of Medicine, University of Washington, Seattle, WA, USA.

**Keywords:** attention-deficit/hyperactivity disorder, problematic smartphone use, domestic violence, parenting styles, peer bullying victimization

## Abstract

**Background:** Problematic smartphone use (PSU) was associated with the increased risk of mental health problems in adolescents. Studies have identified several individual factors related to PSU in adolescents with ADHD; however, environmental factors related to PSU in adolescents with ADHD have not been examined. This cross-sectional questionnaire-survey study examined the associations of domestic violence, parenting styles, and peer bullying victimization with the severity of PSU in adolescents with attention-deficit/hyperactivity disorder (ADHD).

**Methods:** In total, 247 adolescents with ADHD and their parents participated in the study. The severity of PSU was assessed using the Smartphone Addiction Inventory. Domestic violence was assessed using the Parent-to-Child Violence Questionnaire and Violence among Adult Family Members Questionnaire. Parenting styles were assessed using the Parental Bonding Instrument. Peer bullying victimization was assessed using the School Bullying Experience Questionnaire.

**Results:** Violence among adult family members (*p* = .049) and being a victim of social and verbal bullying (*p* = .049) significantly correlated with higher PSU. Authoritarian and controlling parenting significantly correlated with PSU in bivariable but not multivariable regression analysis (*p* > .05).

**Conclusion:** Environmental factors significantly correlated with PSU in adolescents with ADHD. Health professionals should incorporate these factors into the intervention programs for PSU among adolescents with ADHD.

## 1. Introduction

Smartphones have become one of the important devices for adolescents in modern life. Adolescents use smartphones to connect with others, get messages, have fun, and learn; adolescents can explore a wide range of values and develop an independent and liberated self-identity 1. However, smartphones can provide quick fun and close social interaction, which may lead to an increasing dependence on smartphones. Adolescents who have problematic smartphone use (PSU) experience compulsive smartphone use, tolerance to smartphone use, withdrawal symptoms if smartphones are unavailable, and functional impairment due to PSU 2. A meta-analysis found that the median prevalence of PSU amongst children and adolescents was 23.3% 3; PSU was associated with the increased risk of depression, anxiety, perceived stress, and poorer sleep quality 3. The results of studies indicate that PSU in adolescents is an important health issue and warrants further study.

Studies have demonstrated the significant associations of PSU with the diagnosis of ADHD and ADHD symptoms. A study in South Korean adolescents found that adolescents with ADHD have a higher risk of PSU than did those without ADHD (odds ratio = 6.43) 4. PSU was also significantly associated with ADHD symptoms in children 5 and university students 6. PSU can increase psychological distress and then compromise quality of life in individuals with ADHD 7. Several individual factors such as low emotional intelligence 8, difficulty in stress management 8, high boredom proneness 9, aversion for delayed reward 10, high fun seeking 11, low frustration tolerance 11, comorbid oppositional defiant disorder (ODD) and conduct problems 12, comorbid depression and anxiety 13, and low self-esteem 13 have been proposed to explain the association between PSU and ADHD. However, according to Bronfenbrenner's ecological system theory 14, both personal and environmental factors contribute to the development of online compulsive shopping behaviors in adolescents. The environmental factors related to PSU in adolescents with ADHD have not been examined yet.

Domestic violence, parenting styles, and peer bullying victimization are environmental factors that have profound influences on adolescents' mental health and behaviors. According to Bronfenbrenner's ecological system theory 14, these three factors exist in microsystem that has direct contacts with adolescents. The interactions the adolescents have with their family members and peers directly impact adolescents' development. Studies have confirmed that children and adolescents with ADHD have higher exposure of domestic violence compared with children without ADHD 15, 16; ADHD and exposure to domestic violence to have an additive effect on adolescents' aggression and suicide attempts 16. It is possible that domestic violence reduces parents' communication with children and monitoring children's smartphone use. Domestic violence may also cause adolescents to turn to smartphone use for stress relief and interpersonal support. However, the association between domestic violence and PSU in adolescents with ADHD remain unclear. It is hypothesized that:

**Hypothesis (H) 1: Domestic violence is positively and significantly associated with PSU in adolescents with ADHD.**

Parenting style is a key adolescent-parent interaction factor that substantially affects adolescent behavior. Adolescents with ADHD experience unique parenting styles 17. A meta-analysis revealed that positive parenting styles were significantly negatively associated with problematic internet use among adolescents 18. Furthermore, a review demonstrated that adolescents from authoritative households consistently exhibited more protective behaviors and engaged in fewer risk behaviors compared with those from nonauthoritative households 19, whereas permissive and authoritarian parenting styles were positively correlated with internet addiction level of adolescents 20, 21. Despite these findings, the specific associations between different parenting styles, such as caring or affectionate parenting, authoritative parenting, and parenting, and PSU among adolescents with ADHD warrants further investigation. It is hypothesized that:

**H2a: Caring/affectionate parenting are negatively and significantly associated with PSU in adolescents with ADHD.**

**H2b: Authoritative and overprotective parenting is positively and significantly associated with PSU in adolescents with ADHD.**

Studies have found that a high proportion of adolescents with ADHD experience peer bullying victimization 22-24. Bullying victimization causes emotional problems, compromise quality of life, and increase the risk of suicidal ideation in adolescents with ADHD 25, 26. The victimized adolescents may seek interpersonal support and entertainment from smartphones to reduce distress. However, the association between bullying victimization and PSU in adolescents with ADHD has not been examined. It is hypothesized that:

**H3: Bullying victimization is positively and significantly associated with PSU in adolescents with ADHD.**

This cross-sectional questionnaire-survey study examined the associations of domestic violence, parenting styles, and peer bullying victimization with the severity of PSU in adolescents with ADHD. The hypotheses of this study were described above.

## 2. Methods

### 2.1. Participants and procedures

This was a cross-sectional questionnaire survey study. The study participants were adolescents with ADHD and their parents who mainly took care of adolescents. This study enrolled adolescents with ADHD from six child psychiatry outpatient clinics of two hospitals in Taiwan. The inclusion criteria for adolescents with ADHD were as follows: (1) age 11-18 years and (2) having received a diagnosis of ADHD by a certified child psychiatrist in accordance with the Diagnostic and Statistical Manual of Mental Disorders, Fifth Edition (DSM-5) 27. Adolescents and parents who had comorbid intellectual disability, severe autism spectrum disorder, bipolar disorder, schizophrenia, or any other cognitive deficit that could impede their understanding of the study purposes and completion of the research questionnaire were excluded.

Three child psychiatrists reviewed the medical records of adolescents with ADHD who visited the outpatient clinics between August 2023 and July 2024. Subsequently, at the outpatient clinics, we consecutively approached 259 adolescents with ADHD and their parents who met the inclusion criteria. The child psychiatrists interviewed the adolescents and their parents and excluded 12 adolescents with ADHD because they had comorbid autism spectrum disorder (n = 6) and intellectual disability (n = 6). The child psychiatrists explained the study purposes and procedures to the remaining adolescents and their parents and invited them to participate in the study. They were assured that their responses would remain confidential, and that their participation or nonparticipation would not influence their right to receive medical services. In total, 247 adolescents (41 girls and 206 boys, mean age [SD] = 13.2 [2.0] years) with ADHD and their parents (182 females and 65 males, mean age [SD] = 46.4 [6.4] years) agreed to participate in the study.

### 2.2. Ethics statement

This study was approved by the Institutional Review Boards of two university affiliated hospitals. Informed consents were obtained from all adolescents and their parents involved in the study. This questionnaire-survey study did not apply any experiments on humans or the use of human tissue samples. This paper conforms to the Declaration of Helsinki and Recommendations for the Conduct, Reporting, Editing, and Publication of Scholarly Work in Medical Journals.

### 2.3. Measures

#### 2.3.1. Smartphone addiction inventory

The 26-item Smartphone Addiction Inventory (SPAI) was used to assess the participants' self-reported severity of PSU in the one year prior to the assessments 2. The participants rated each item on a 4-point scale ranging from 1 (totally disagree) to 4 (totally agree), with a total score ranging from 26 to 104. A higher total score indicated a higher level of PSU. The SPAI has acceptable reliability and validity in the original study 2. The Cronbach's α coefficient of the SPAI in the present study was .92.

#### 2.3.2. Rosenberg self-esteem scale

This study used the 10-item Rosenberg Self-Esteem Scale (RSES) to evaluate adolescents' self-reported self-esteem 28. Each item was rated on a four-point scale response scale ranging from 1 (strongly disagree) to 4 (strongly agree). The scale yields a single overall score of self-esteem, with high scores indicating high levels of self-esteem. This scale has been previously used to evaluate the level of self-esteem in Taiwanese adolescents 29. Cronbach's a in the present study was .860.

#### 2.3.3. Child behavior checklist for ages 6-18

The 112-item parent-reported Chinese version of the Child Behavior Checklist for Ages 6-18 (CBCL/6-18) was used to measure adolescents' behavior problems 30, 31. We used the recommended T-score transformations of raw behavior scores, which were adjusted for age and sex differences in behavior found in normative samples. We used the domains of ADHD problems, internalizing problems (which includes scales for anxiety/depression, withdrawal/depression, and somatic complaint syndrome) and externalizing problems (which includes scales for oppositional defiant disorder and conduct symptoms) for analysis. The internal consistency (Cronbach α) ranges from .55 to .90, one-month test-retest reliability (Pearson *r*) ranges from .51 to .74, and construct validity (eight-factor structure) have been demonstrated 32, 33.

#### 2.3.4. Parental bonding instrument

The 25-item Chinese version of the Parental Bonding Instrument (PBI)-parent version was used to evaluate the parents' perceptions of three parenting styles: caring or affectionate parenting, authoritative parenting, and overprotective parenting 34. Each item is rated on a 4-point Likert scale. A high score on the care/affection subscale reflects parents' perceptions of parental warmth and affection, whereas a low score indicates perceptions of rejection or indifference. The overprotection subscale measures overprotective parenting behaviors and denial of adolescents' psychological autonomy. The authoritarianism subscale evaluates the degree of authoritative control that parents exert over adolescents' behavior 35. The reliability and validity of the Chinese version of the PBI were established in a previous study 36. In this study, the Cronbach's α values were .78 for caring or affectionate parenting, .70 for overprotective parenting, and .68 for authoritative parenting, indicating acceptable internal consistency.

#### 2.3.5. Domestic violence

This study assessed two forms of domestic violence, including parent-to-child violence and violence among adult family members. This study adopted seven items from the Child-to-parent Violence Questionnaire 37 to develop the child-reported Parent-to-Child Violence Questionnaire (PCV-Q) and parent-reported Violence among Adult Family Members Questionnaire (VAFM-Q). The PCV-Q assessed parents' verbal (four items) and physical violence (3 items) to adolescents in the preceding year. The VAFM-Q assessed verbal and physical violence among adult family members in the preceding year. The items in both questionnaires were rated on a five-point scale same as the CDPV-Q. Internal consistency (McDonald's ω) of the PCV-Q and CDPV-Q was .76 to .72, respectively. The answers other than 0 to the items of the PCV-Q and CDPV-Q indicate having parent-to-child violence and violence among adult family members.

#### 2.2.6. Chinese version of the school bullying experience questionnaire

The self-reported Chinese version of the School Bullying Experience Questionnaire (C-SBEQ) was used to evaluate participants' experiences of peer bullying victimization at schools and cram schools in the previous year. Eight items assessing the experiences of victimization of social and verbal bullying (four items) and physical bullying (four items) were answered on a 4-point Likert scale 38, 39. The C-SBEQ has acceptable reliability and validity 39. In this study, the Cronbach's α values were .81 for victimization of social and verbal bullying and 0.67 for victimization of physical bullying. Participants who answered 2 or 3 on any item among items 1 to 4 and items 5 to 8 were identified as self-reported victims of social and verbal bullying and physical bullying, respectively.

#### 2.3.7. Demographic characteristics

Adolescents' gender and age and parents' gender, age, and education level were collected.

### 2.4. Data analysis

Statistical analyses were performed using IBM SPSS Statistics version 24.0 (IBM Corporation, Armonk, NY, USA). Descriptive statistics (presented as means and frequencies) were used to summarize the characteristics of the study sample. Associations of individual factors (adolescent demographics, self-esteem, parent's education level, ADHD problems, internalizing problems, and externalizing problems), family factors (parenting styles and domestic violence), and peer factor (peer bullying victimization) with PSU were firstly examined using bivariable linear regression analysis. The associations of parenting styles, domestic violence, and peer bullying victimization with PSU were examined using multivariable linear regression analysis in separate models. A *p* value < .05 was considered to indicate statistical significance.

## 3. Results

Participants' demographics, adolescents' behavioral problems, self-esteem, parents' parenting styles, domestic violence, peer bullying victimization, and PSU are shown in Table 1. The mean score of the SPAI was 42.6 (SD = 16.0). All values of skewness and kurtosis of continuous variables ranged between -1 and 1, indicating that these continuous variables were normally distributed.

The results of examining the factors correlated with PSU using bivariable linear regression analysis are shown in Table 2. Older age (p < .001) and being a victim of social and verbal bullying (p = .035) were significantly associated with higher PSU. High self-esteem (p < .001), higher authoritarian and controlling parenting (p = .049), and violence among adult family members (p = .024) were significantly associated with lower PSU. Adolescent gender, ADHD problems, parent's education level, internalizing and externalizing behavioral problems, affectionate and overprotective parenting, parent-to-child violence and being a victim of physical bullying were not significantly associated with PSU (all p > .05).

The results of examining the associations of parenting styles, domestic violence, and peer bullying victimization with PSU are shown in Table 3. The results of Model I demonstrated that older age (*p* < .001) and lower self-esteem (*p* < .001) significantly correlated with PSU. The results of Model II demonstrated that after adjusting the effects of individual factors, parenting styles did not significantly correlate with PSU (all *p* > .05). The results of Model II demonstrated that after adjusting the effects of individual factors, parenting styles did not significantly correlate with PSU (all *p* > .05). The results of Model III demonstrated that violence among adult family members significantly correlated with PSU (*p* = .049), whereas parent-to-child violence did not significantly correlate with PSU (*p* > .05). The results of Model IV demonstrated that being a victim of social and verbal bullying significantly correlated with PSU (*p* = .049), whereas being a victim of physical bullying did not significantly correlate with PSU (*p* > .05).

## 4. Discussion

The present study found that several environmental factors in microsystem significantly correlated with PSU in adolescents with PSU. Being a victim of social and verbal bullying and violence among adult family members were significantly associated with higher PSU. Authoritarian and controlling parenting significantly correlated with PSU in bivariable but not multivariable regression analysis.

Victimization of social and verbal bullying is a distressing experience for adolescents. For adolescent bullying victims, there is a strong association between bullying victimization and psychological symptoms (e.g., depression, difficulties in getting to sleep, and loneliness, helplessness) 25-27, 40. The victimized adolescents may use their smartphones for entertainment such as listening to music, watching videos, and playing games to improve their mood. A meta-analysis found a significant association between internet gaming disorder and bullying victimization in adolescents 41. The victimized adolescents may seek interpersonal interactions via smartphones to compensate for interpersonal difficulties caused by real-life bullying. However, PSU may increase the risk of cyberbullying victimization in adolescent 42. Studies have also found that adolescents with ADHD are more likely to suffer from both victimization in cyberbullying and traditional bullying 43, 44. Victimization of social and verbal bullying and PSU may form a vicious cycle that leads to exacerbating each other's severity. This study did not find a significant association between being a physical bullying victim and PSU in adolescents with ADHD. Only 7.7% of the participants were physical bullying victims in this study; the small number of physical bullying victims might limit the inference of the relationship between being a physical bullying victim and PSU.

The present study found a negative association between authoritarian and controlling parenting and PSU in adolescents with ADHD. Authoritative-authoritarian parenting typologies proposed by Baumrind 45 are important to understand parenting behavior in Western cultures.

However, several studies have found that Authoritative-authoritarian parenting typologies are also applicable to modern Chinese parenting behaviors 46, 47. Authoritarian and controlling parenting reflects the degree of authoritarian-quality parental control over their adolescents' behaviors 35. Parents who adopt an authoritarian attitude expect their children to completely fulfill their orders. It is hypothesized that parents of adolescent with electric device addiction may have an authoritarian parenting to control their child's electric device use 20, 21. Studies have found that authoritarian parenting styles were positively correlated with internet addiction level of adolescents 20, 21. However, the result of this study was different from those of previous studies. It is hypothesized that authoritative-authoritarian parenting typologies may not be the same concept in both Western and Chinese cultures. For example, under the cultivation of individualism, Western societies emphasize the positive development of children's personalities, thus aiming to cultivate independence, creativity and diversity; whereas traditional Chinese societies emphasize familialism, and the main focus of the parenting philosophy is to raise children who meet the expectations of society, and to make the child a person who can honor the family and shine 48. In this way, the authoritarian and controlling parenting of parents in traditional Chinese societies may be consistent with the discipline of children's smartphone use for the sake of children's academic achievement and then reduce the severity of PUS. Further, studies have found the specific aspects of parenting in Chinese parents, such as “*guan*” 49, 50. G*uan* taps into the sense of responsibility endorsed by Chinese parents in their childrearing. Central to this responsibility is that parents govern and train children through providing close monitoring, firm directives, and high demands to help children develop into well-funding members of society 49. Intriguingly, the concepts of *guan* are positively related to both authoritative and authoritarian parenting 51, 52. From the above findings, investigating both Authoritative-authoritarian parenting typologies and Chinese-specific parenting (e.g., “*guan*”) simultaneously will help to understand the relationship between parenting behaviors and PSU among adolescents in contemporary Chinese-cultural societies.

Violence among adult family members significantly correlated with PSU after adjusting the effects of individual factors. Violence among adult family members will result in parents having no time to care for their children and control their behaviors and increase the risk of adolescents' PSU 53. Domestic violence may also compromise adolescents' self-control and friendship quality and then increase PSU 54. Domestic violence may also increase the risk of electric device addiction through emotional and sleep problems 55, 56. However, the association between violence among adult family members and PSU became insignificant in multivariable regression analysis. Given that exposure to domestic violence was significantly associated with lower self-identity development 57, the association between violence among adult family members and PSU might be confounded by self-esteem.

The present study did not find the significant association between parent's education level and PSU in adolescents with ADHD. However, we did not examine the roles of other socioeconomic factors and community environment such as the families', schools' and communities' attitudes toward adolescent smartphone use for PSU. These factors existing in microsystem, mesosystem, and exosystem may influence adolescents' smartphone use. The correlations of these factors with PSU in adolescents with ADHD warrant study in future. Further qualitative studies such as interviews or focus groups with adolescents and parents also help to provide insights into the experiences and perceptions surrounding PSU. The present study has several limitations. First, adolescents with ADHD were recruited from outpatient clinics, where they were actively receiving pharmacological or psychological therapy. Future studies should investigate whether the study findings can be extrapolated to adolescents with ADHD who are not receiving medical treatment and TD adolescents recruited through alternative methods. Including a more diverse sample population across different cultural and socioeconomic backgrounds could provide a broader understanding of PSU in adolescents with ADHD. Second, given the cross-sectional design of the present study, the temporal associations between PSU and other variables could not be determined. Third, this study did not assess smartphone usage types (e.g., educational vs. recreational). Research found that social network service and music/videos positively correlated with PSU, whereas study negatively correlated with PSU 58.

## 5. Conclusion

The present study found that being a victim of social and verbal bullying was significantly associated with higher PSU in adolescents with ADHD. It is necessary to survey the existence of PSU among adolescents with the experience of bullying victimization. Health professionals should understand the relationship between bullying experiences and PSU in adolescents and help develop strategies to control smartphone use. Authoritarian and controlling parenting and violence among adult family members significantly correlated with PSU in bivariable regression analysis. Although the correlations became nonsignificant in multivariable regression analysis, parenting styles and domestic violence are environmental factors that warrants survey in managing PSU among adolescents with ADHD. Several intervention programs such as cognitive-behavioral therapy, enhancing family support and supervision, changing lifestyle, use of assistive technology, exercise, mindfulness, and meditation have been proposed for PSU 59. Health professionals should help adolescents and parents based on these intervention models and integrate the interventions of parenting styles, domestic violence, and bullying into the models.

## Figures and Tables

**Table 1:** Demographic, Behavioral Problems, Self-esteem, Parenting Styles, Domestic Violence, and Peer Bullying Victimization (N = 247)

	*n* (%)	Mean (SD)	Range
Adolescent gender			
Girls	41 (16.6)		
Boys	206 (83.4)		
Adolescent age (years)		13.2 (2.0)	11-18
Parent gender			
Females	182 (73.7)		
Males	65 (26.3)		
Parent age (years)		46.4 (6.4)	27-76
Parent education level			
High school or below	80 (32.4)		
College or above	167 (67.6)		
Behavioral problems on the CBCL/6-18			
ADHD problems		61.9 (7.7)	50-80
Internalizing behavior problems		56.8 (10.2)	33-85
Externalizing behavior problems		55.7 (10.3)	33-78
Self-esteem on the RSES		19.2 (6.0)	3-30
Parenting styles on the PBI			
Affectionate parenting		38.9 (4.8)	23-48
Overprotective parenting		12.4 (3.0)	7-20
Authoritarian and controlling parenting		11.7 (2.9)	6-20
Parent-to-child violence	96 (38.9)		
Violence among adult family members	102 (41.3)		
Social and verbal bullying victims	61 (24.7)		
Physical bullying victims	19 (7.7)		
Problematic smartphone use on the SPAI		42.6 (16.0)	26-96

ADHD: attention-deficit/hyperactivity disorder; CBCL/6-18: Child Behavior Checklist for Ages 6-18; PBI: Parental Bonding Instrument; RSES: Rosenberg Self-Esteem Scale; SPAI: Smartphone Addiction Inventory

**Table 2:** Factors Correlated With Problematic Smartphone Use: Bivariable Linear Regression Analysis

	Unadjusted *B* (se)	95% CI of *B*
Adolescent gender^a^	-2.528 (2.739)	-7.922, 2.867
Adolescent age	2.352 (0.482)***	1.403, 3.300
Parent's education level at college or above^ b^	-1.444 (2.179)	-5.737, 2.849
ADHD problems	0.110 (0.132)	-0.150, 0.370
Internalizing behavioral problems	0.150 (0.100)	-0.047, 0.347
Externalizing behavioral problems	0.032 (0.099)	-0.163, 0.227
Self-esteem	-0.756 (0.165)***	-1.081, -0.432
Affectionate parenting	0.233 (0.214)	-0.188, 0.655
Overprotective parenting	-0.013 (0.345)	-0.692, 0.666
Authoritarian and controlling parenting	-0.701 (0.355)*	-1.400, -0.001
Parent-to-child violence	2.916 (2.086)	-1.192, 7.025
Violence among adult family members	-4.669 (2.052)*	-8.711, -0.628
Social and verbal bullying victims	4.985 (2.346)*	0.365, 9.605
Physical bullying victims	1.491 (3.830)	-6.052, 9.034

^a^: Girls as the reference; ^b^: High school or below as the reference ADHD: attention-deficit/hyperactivity disorder; CI: confidence interval; se: standard error *p: < .05; ***p: < .001

**Table 3:** Factors Correlated With Problematic Smartphone Use: Multivariable Linear Regression Analysis

	Model I		Model II		Model III		Model IV	
	Adjusted *B* (se)	95% CI of *B*	Adjusted *B* (se)	95% CI of *B*	Adjusted *B* (se)	95% CI of *B*	Adjusted *B* (se)	95% CI of *B*
Adolescent gender^a^	0.424 (2.652)	-4.801, 5.649	0.250 (2.644)	-4.958, 5.459	0.480 (2.662)	-4.764, 5.725	1.261 (2.686)	-4.030, 6.552
Adolescent age	2.163 (0.495)***	1.188, 3.138	2.183 (0.495)***	1.208, 3.158	2.079 (0.495)***	1.105, 3.054	2.317 (0.499)***	1.334, 3.301
Parent's education level at college or above^b^	-1.490 (2.053)	-5.535, 2.554	-1.218 (2.070)	-5.296, 2.860	-1.277 (2.050)	-5.315, 2.761	-1.597 (2.047)	-5.629, 2.435
ADHD problems	0.218 (0.184)	-0.144, 0.580	0.222 (0.187)	-0.147, 0.591	0.227 (0.184)	-0.136, 0.589	0.203 (0.185)	-0.160, 0.567
Internalizing behavioral problems	0.024 (0.125)	-0.222, 0.269	0.026 (0.127)	-0.223, 0.276	0.041 (0.124)	-0.204, 0.285	0.034 (0.124)	-0.211, 0.279
Externalizing behavioral problems	-0.046 (0.156)	-0.353, 0.260	-0.002 (0.157)	-0.310, 0.307	-0.015 (0.156)	-0.324, 0..293	-0.053 (0.155)	-0.359, 0.252
Self-esteem	-0.624 (0.169)***	-0.957, -0.290	-0.639 (0.171)***	-0.976, -0.301	-0.567 (0.174)**	-0.910, -0.225	-0.561 (0.174)**	-0.902, -0.219
Affectionate parenting			0.385 (0.215)	-0.038, 0.808				
Overprotective parenting			0.215 (0.336)	-0.446, 0.876				
Authoritarian and controlling parenting			-0.343 (0.368)	-1.068, 0.381				
Parent-to-child violence					1.325 (2.106)	-2.824, 5.474		
Violence among adult family members					-4.120 (2.080)*	-8.218, -0.022		
Social and verbal bullying victims							4.573 (2.359)*	0.002, 9.220
Physical bullying victims							-2.715 (3.722)	-10.048, 4.617
Adjusted R^2^	0.130		0.138		0.137		0.137	

^a^: Girls as the reference; ^b^: High school or below as the reference se: standard error *p: < .05; **p: < .01; ***p: < .001
